# Estimation of individual tree aboveground biomass of genetically diverse *Catalpa bungei* based on nonlinear mixed-effects models and UAV LiDAR data

**DOI:** 10.3389/fpls.2026.1759637

**Published:** 2026-03-09

**Authors:** Xinru Fu, Wenjun Ma, Qiao Chen, Liyong Fu, Miaomiao Zhang, Guangshuang Duan, Yang Zhang, Ziyan Zheng, Chuangye Wu, Qingqing Wang, Yuheng Shun, Pan Li

**Affiliations:** 1 Institute of Forest Resource Information Techniques, Chinese Academy of Forestry, Beijing, China; 2 Key Laboratory of Forest Management and Growth Modelling, National Forestry and Grassland Administration, Beijing, China; 3 Chinese Academy of Forestry Research, Institute of Forestry, Beijing, China; 4 State Key Laboratory of Tree Genetics and Breeding, Key Laboratory of Tree Breeding and Cultivation of State Forestry Administration, Beijing, China; 5 Xinyang Normal University, Xinyang, Henan, China; 6 Wen County Institute of Forestry Science, Jiaozuo, Henan, China

**Keywords:** aboveground biomass, *Catalpa bungei*, genotypic variation, LiDAR, UAV, nonlinear mixed-effects model

## Abstract

**Introduction:**

Accurate estimation of individual tree aboveground biomass (AGB) is essential for tree species selection, carbon accounting, and precision forestry. Unmanned aerial vehicle (UAV) LiDAR provides rapid access to detailed tree structural information, offering a promising tool for high-frequency biomass assessment.

**Methods:**

In this study, a nonlinear mixed-effects (NLME) model integrating UAV LiDAR and field measurements was developed to quantify the influence of genetic heterogeneity and environmental factors on AGB estimation of *Catalpa bungei*. Data from 2,941 trees across 79 genotypes were collected in Henan Province, including LiDAR-derived tree height (*L_H_*), LiDAR-derived crown diameter (*L_CD_*), and AGB. By incorporating genotype as a random effect and planting density as a dummy variable, the NLME model significantly outperformed traditional dummy-variable models.

**Results:**

Genotype effects explained significant AGB variation, achieving high accuracy (R²=0.7916, RMSE = 3.7095) and reducing TRE by 23.29% compared to the basic power function model. Leave-one-genotype-out cross-validation confirmed robustness. Calibration with the four largest trees yielded the best performance (TRE = 13.09%), while a simplified scheme using only two trees per genotype maintained high accuracy (TRE = 13.24%), markedly reducing field effort.

**Discussion:**

These results highlight the superiority of NLME AGB models over linear approaches and demonstrate that accounting for genotype effects is critical for reliable biomass estimation. The proposed framework provides an efficient and cost-effective solution for biomass monitoring, tree breeding, carbon sink assessment, and precision forestry.

## 1 Introduction

Forests are a major component of terrestrial ecosystems and play an important role in maintaining ecological stability, regulating the global carbon cycle, and mitigating climate change (Hyyppa et al., 2008). Biomass is a key biophysical variable for quantifying terrestrial carbon stocks and dynamics and is widely used in ecological studies, forest management planning, and assessments of forest growth and carbon cycling (Dixon, 1994; Jenkins et al., 2003). However, traditional biomass estimation methods based on tree height and diameter at breast height (DBH) rely heavily on field measurements, which are labor-intensive, time-consuming, and costly. Improving the efficiency and accuracy of biomass estimation therefore remains a central challenge in forest resource monitoring and sustainable forest management (Dai and Yu, 2004; Fu et al., 2016). To address these limitations, remote sensing technologies have been increasingly applied to forest biomass estimation. Data acquired from satellite and airborne platforms enable the monitoring of forest biomass over large spatial extents and extended time periods (Han et al., 2019; Puletti et al., 2020; Gonçalves et al., 2023).

Light detection and ranging (LiDAR) is an active remote sensing technology that characterizes forest three-dimensional structure by emitting laser pulses and recording their returns. Among existing remote sensing approaches, LiDAR systems mounted on unmanned aerial vehicles (UAVs) can generate dense three-dimensional point clouds at the stand scale, providing high-resolution structural information that captures both vertical and horizontal forest structure and enables detailed characterization of individual trees. Structural metrics derived from UAV-LiDAR data, such as tree height and crown width, are strongly correlated with tree biomass and therefore constitute the primary input variables for individual-tree biomass estimation models (Popescu, 2007; Hong et al., 2024). Owing to its high spatial resolution and operational flexibility at plot and stand scales, UAV LiDAR has been widely applied in individual-tree biomass modeling (Pang and Li, 2012; Tian et al., 2023).

*Catalpa bungei C. A. Mey.*, a broad-leaved tree species in the family Bignoniaceae, is an important timber and ornamental species in China. It is widely distributed across the Yellow River and Yangtze River basins and is valued for its roles in windbreaks, sand stabilization, timber production, landscaping, and environmental protection. Existing studies on *Catalpa bungei* have mainly focused on variety breeding (Zheng et al., 2017; Zhao et al., 2020), clonal propagation techniques (Hu Q. et al., 2021; Mou et al., 2023), growth characteristics (Qiao et al., 2024; Zhao et al., 2025), and germplasm conservation and utilization (Li et al., 2019; Yang et al., 2020). Biomass-related studies have mostly examined biomass allocation patterns (Zhang et al., 2021; Guan et al., 2024; Chen et al., 2025), whereas relatively few studies have focused on developing biomass inversion models at the individual-tree level. Developing robust individual-tree biomass estimation models for *Catalpa bungei* is therefore essential for genotype selection, forest management, and accurate carbon stock assessment.

Aboveground biomass (AGB) is a widely used indicator of forest ecosystem productivity. Since at least the 17th century, attempts to quantify forest resources have been documented in forest science (e.g., John Evelyn’s Sylva, 1664), providing an early conceptual basis for modern forest biomass research. From a methodological perspective, remote sensing–based AGB estimation approaches can be broadly classified into traditional allometric models, machine learning–based models, and mixed-effects models. Traditional allometric models are simple and interpretable but typically require extensive field data and assume homogeneous growth responses (Jenkins et al., 2003). Machine learning methods, such as random forests and support vector machines, have been increasingly applied to biomass estimation based on lidar due to their ability to simulate complex nonlinear relationships. However, they usually lack biological interpretability and provide limited support for hierarchical calibration or genotype-level inferences (Breiman, 2001; Cutler et al., 2007).

Dummy-variable models represent categorical factors as fixed effects using binary indicators, providing a simple and interpretable way to account for systematic differences among discrete groups, such as planting density classes (Sharma et al., 2019). However, because these effects are treated as fixed, dummy-variable models alone cannot capture continuous biological variability, which motivates their integration with mixed-effects frameworks in hierarchical AGB analyses (Zhu et al., 2025). Mixed-effects models incorporate both fixed effects, describing population-level relationships, and random effects, representing group-specific deviations arising from hierarchical data structures, and are therefore well suited to forestry data that are commonly nested and spatially correlated (Pinheiro and Bates, 2000). Nonlinear mixed-effects (NLME) models further extend this framework by allowing nonlinear predictor-response relationships while retaining both fixed and random effects, providing a more realistic representation of biological growth processes and allometric relationships (Calama and Montero, 2004).

In recent decades, biomass modeling has further advanced through the application of mixed-effects models that account for hierarchical data structures and variability across spatial or biological units (Picard et al., 2012). For instance, Li et al. employed a NLME model to simulate stem cumulative biomass of *Larix gmelinii* in northeastern China, accounting for tree-level random effects. Their findings showed that the NLME model outperformed models fitted by ordinary least squares in prediction accuracy (Li et al., 2011). Xu Zhe et al. developed a biomass model for *Pinus armandii* forests using an NLME approach, incorporating regional plot effects as random effects and accounting for variance-covariance structures. The model achieved a prediction accuracy of 77.83% (Xu et al., 2017). Xie Dongbo et al. combined NLME models with various machine learning techniques to predict shrubland AGB in arid regions, considering plot density and intensity as variables. They found that support vector machines yielded the highest prediction accuracy, and the mixed-model approach significantly outperformed traditional baseline models (Xie et al., 2023). Despite the increasing application of mixed-effects and remote sensing–based AGB models, most existing studies primarily focus on environmental heterogeneity (e.g., plot or site effects) or operate at the species level, while genetic heterogeneity among tree genotypes is rarely incorporated. This limitation is particularly relevant for multi-genotype plantations, where genotype-driven differences in tree architecture can introduce systematic bias if not explicitly modeled. Treating genotype as a random effect therefore provides a biologically meaningful and statistically robust way to disentangle genetic and environmental sources of variation, while also enabling efficient model calibration under limited field sampling conditions (Song et al., 2022).

Although UAV LiDAR–based biomass estimation has shown strong potential, its large-scale application remains constrained by the cost of field data collection. Compared with airborne and satellite LiDAR systems, UAV platforms are constrained in spatial coverage by battery endurance and flight regulations, highlighting the need for efficient calibration and sampling strategies in operational applications. Optimizing sampling strategies to reduce field effort while maintaining predictive accuracy is therefore important for operational forestry. Developing a modeling framework that achieves reliable biomass estimation with only a small number of calibration trees per genotype would substantially improve the efficiency of AGB monitoring and carbon accounting.

In this study, we utilize UAV LiDAR data in combination with ground-based measurements to develop a systematic and robust modeling framework for individual-tree AGB estimation of *Catalpa bungei*. The proposed approach incorporates planting density as a dummy variable and genotype as a random effect, explicitly accounting for both environmental variability and genetic heterogeneity. In addition, we evaluate optimized field calibration strategies to reduce sampling effort while maintaining robust predictive performance. Rather than replacing machine-learning approaches, the proposed NLME framework provides an interpretable, calibration-friendly alternative that explicitly represents hierarchical structure and genotype-level variability. The proposed approach provides a quantitative basis for genotype-aware forest carbon assessment and supports large-scale biomass monitoring, tree breeding evaluation, and precision forest management. The primary objectives of this study are: 1) to develop a UAV LiDAR–based NLME AGB model for *Catalpa bungei* that explicitly incorporates genotype as a random effect and planting density as a dummy variable, thereby accounting for genetic heterogeneity and environmental variability; 2) to evaluate optimized field calibration strategies that minimize sampling effort while maintaining robust predictive performance for large-scale applications; 3) to establish a genetic-aware biomass modeling framework that improves the consistency, interpretability, and practical applicability of LiDAR-based AGB estimation, providing methodological support for precision forestry and genotype-specific carbon accounting.

## 2 Materials and methods

### 2.1 Study area

The study area is located in Wen County, Jiaozuo, Henan Province, China (112°51′–113°1′ E, 34°52′–35°2′ N), at an elevation of 102.3–116.1 m. The average annual temperature is 14–15°C, and annual precipitation ranges from 550 to 700 mm. The region has a warm temperate continental monsoon climate.

**Figure 1:**
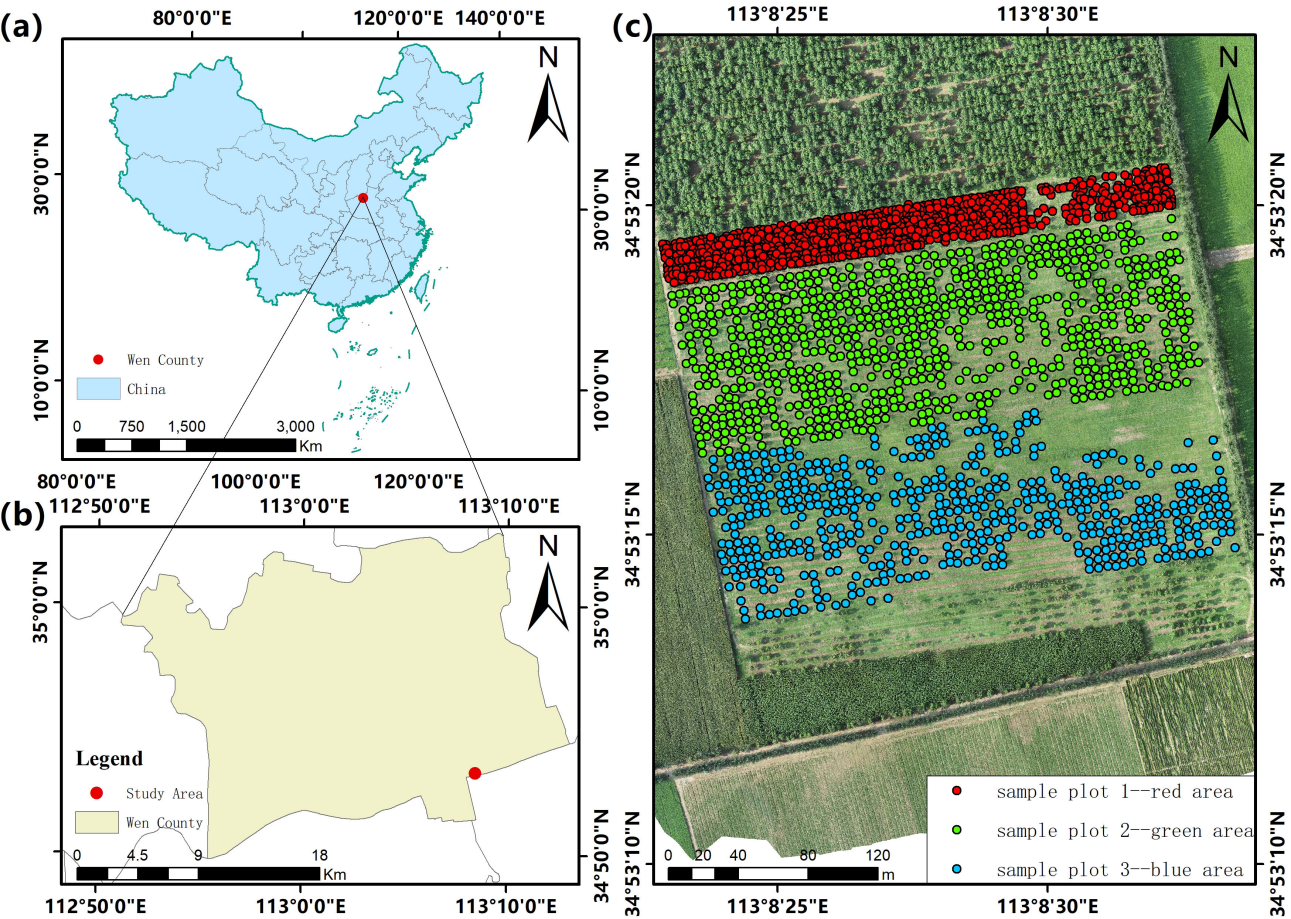
Overview of the study area and experimental design. **(a)** Location of the study area in China. **(b)** Administrative boundary of Wen County indicating the experimental site. **(c)** UAV-derived orthomosaic image showing the layout of three experimental plots (red, green, and blue areas).

The *Catalpa bungei* trees in the study area were planted in the spring of 2020 as one-year-old seedlings, covering an area of 130 mu (approximately 8.7 hectares; see **Figure 1**). The area comprises three experimental plots: Red zone (Plot 1): High-density planting with standard fertilization, spacing of 2 m×2 m, arranged in 120 rows and 10 columns, including 200 genotypes with 6 trees per genotype. Green zone (Plot 2): Standard fertilization, spacing of 4 m×4 m, arranged in 60 rows and 20 columns, 200 genotypes with 6 trees per genotype. Blue zone (Plot 3): Nitrogen-deficient fertilization, spacing of 4 m×4 m, arranged in 60 rows and 20 columns, 200 genotypes with 6 trees per genotype. The 200 genotypes were pre-selected at the nursery stage as part of an early-generation breeding program aimed at identifying superior clones for timber and carbon-sequestration traits. Their replication across all three plots allows the mixed-effects model to isolate genetic from environmental influences on biomass. Here, planting density is represented by the fixed inter-tree spacing of 2 m or 4 m that defines the grid within each plot, and it is treated as a plot-level categorical dummy variable.

### 2.2 Data datasets

#### 2.2.1 Data acquisition

The UAV LiDAR data were collected in August 2021, October 2023, and November 2024 using a HuaCe BB4 UAV equipped with an AS-1300HL LiDAR system. UAV LiDAR data were collected concurrently with the field surveys in August 2021, October 2023, and November 2024. The UAV platform used was the BB-4 Bumblebee, equipped with an AS-1300HL LiDAR sensor. A total of four flights were conducted, with an average flight altitude of 80 m, a cruise speed of 6 m/s, and a point cloud density of approximately 350 pts/m². The LiDAR system operated at a wavelength of 1550 nm, with a pulse width of 3.5 ns, a beam divergence of 0.5 mrad, a pulse repetition frequency of 50 kHz, and a maximum scan angle of 30°. The scanning frequency was 49 Hz. Flight lines were arranged in a grid pattern with 50% lateral overlap. The overall average flight speed was 10 m/s, resulting in a final average point cloud density of 110 pts/m² over the study plots.

Field surveys were conducted in the study area in August 2021, October 2023, and November 2024 to measure all *Catalpa bungei* trees with a DBH ≥1 cm. For each sample tree, total height and height to the lowest live branch were measured using a Vertex IV laser hypsometer with a precision of 0.1 m. DBH was measured with a diameter tape to a precision of 0.1 cm. Crown diameter was measured in four directions (east, south, west, and north) using a NOYAFA laser rangefinder, accurate to 0.1 m. The collected data included H, DBH, and CD in four directions (**Table 1**). To ensure model stability and avoid bias from dead trees, only genotypes with more than 30 living samples were used for modeling, resulting in a total of 2,941 trees across 79 genotypes.

**Table 1:** Summary of ground survey data.

Year	Variable	Mean	Max	Min	SD
2021	H (m)	2.01	3.30	1.30	0.44
	DBH (cm)	1.59	3.80	0.50	0.57
	CD (m)	0.64	1.20	0.20	0.14
	AGB (kg)	0.34	2.33	0.02	0.30
2023	H (m)	3.94	6.30	1.70	0.82
	DBH (cm)	5.50	10.00	1.10	1.61
	CD (m)	1.90	3.90	0.40	0.58
	AGB (kg)	5.34	23.08	0.12	4.00
2024	H (m)	5.43	8.20	2.40	1.02
	DBH (cm)	8.41	14.35	2.00	2.05
	CD (m)	2.53	4.35	0.43	0.68
	AGB (kg)	14.52	49.48	0.51	8.85

SD, standard deviation; H, tree height; DBH, diameter at breast height; CD, crown diameter; AGB, aboveground biomass.

The raw data files were imported into Copr 2.0 software for POS solution to generate POS trajectories and LAS point cloud data. LiDAR360 software was used for point cloud preprocessing, including denoising of the acquired point cloud data, ground point classification, height normalization, and individual tree segmentation. Segmentation parameters were set as follows: grid size of 0.25 m, buffer size of 20 pixels, ground point height threshold of 1 m, minimum tree height of 1.5 m, Gaussian smoothing with sigma = 1 and radius = 5 pixels.

#### 2.2.2 Data pre-processing

##### 2.2.2.1 Airborne data processing

The original LiDAR point cloud was first denoised to remove anomalous returns caused by sensor noise, atmospheric effects, and non-vegetation objects, using a combination of height-threshold filtering and density-based outlier detection (Zhao et al., 2024). Ground points were then classified by jointly considering local slope variation, echo characteristics, and neighborhood relationships, allowing robust identification of the ground surface even under complex terrain conditions. Based on the extracted ground points, a high-resolution digital terrain model (DTM) was generated, and the point cloud was normalized by converting elevations to heights above ground, thereby eliminating topographic effects on canopy structure and ensuring consistent tree-height estimation.

##### 2.2.2.2 Individual tree segmentation

**Figure 2:**
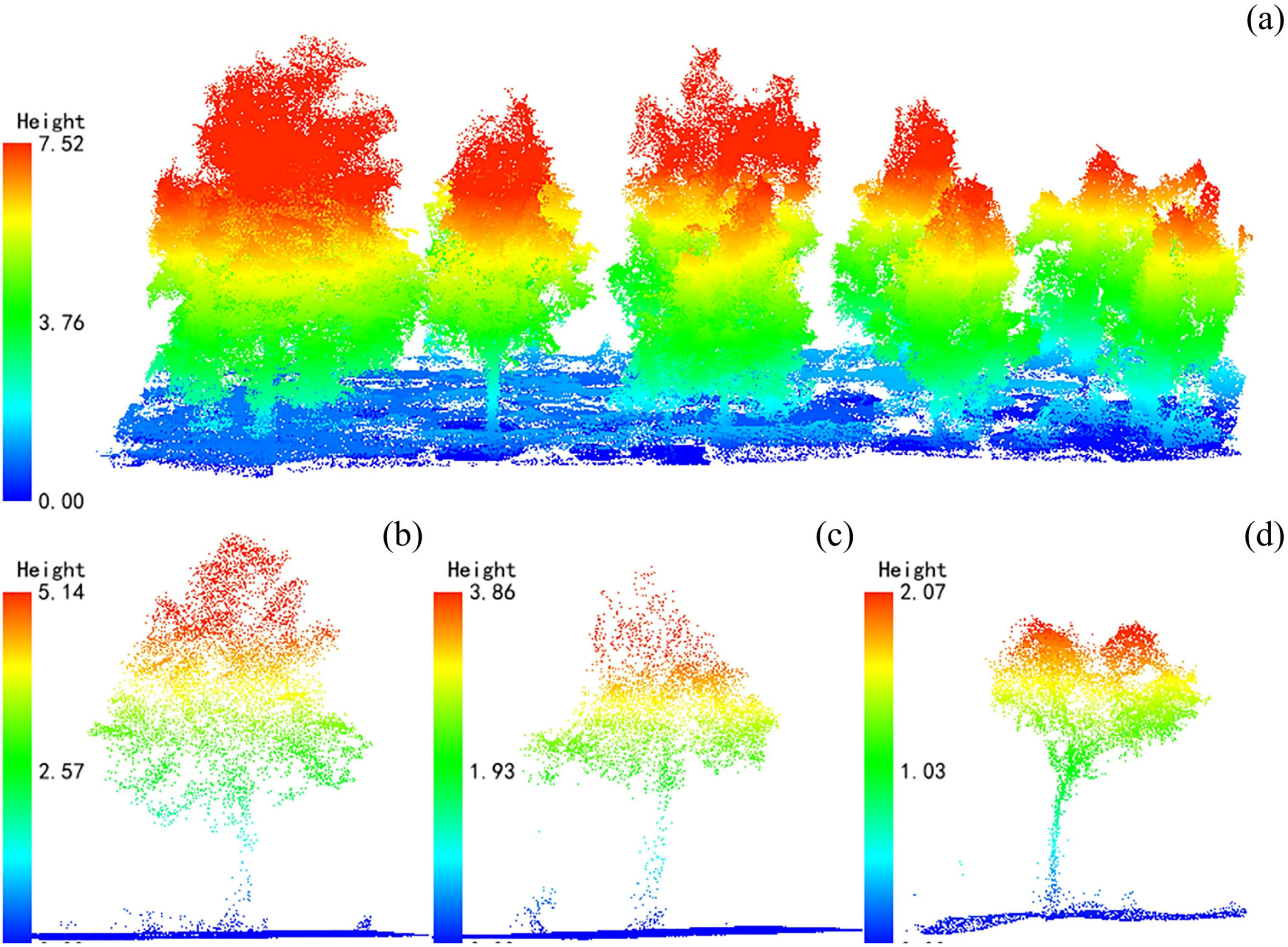
UAV LiDAR point cloud maps of *Catalpa bungei* plantations, showing a schematic view of part of the sample plot **(a)** and mean tree height representations for 2021, 2023, and 2024 **(b–d)**; after height normalization, ground height is set to zero and indicated by blue point clouds, and vegetation height is expressed as aboveground height.

The normalized point cloud was segmented into individual trees using a hybrid approach combining a canopy height model (CHM) and local maxima detection. A CHM raster was first generated, and local height maxima were identified as putative tree apices. These seeds were subsequently expanded into crown segments via marker-controlled watershed or region-growing algorithms, while segmentation parameters were adaptively refined on the basis of local point density, crown width, and height variability. This adaptive strategy markedly improves the accuracy of single-tree detection within heterogeneous forest stands. Finally, LiDAR-derived tree height (*L_H_*) and LiDAR-derived crown diameter (*L_CD_*) were extracted at the individual-tree level. *L_H_* was quantified as the maximum vertical distance between the highest non-ground return and the corresponding ground surface within each segmented crown. *L_CD_* was estimated from the projected crown area using a convex-hull based equivalent circular diameter method (Wang et al., 2023). The UAV-LiDAR point cloud representations of Catalpa bungei plantations across different survey years are shown in **Figure 2**.

#### 2.2.3 Calculation of aboveground biomass of individual trees

Due to the requirements of *Catalpa bungei* propagation and clonal selection, destructive sampling was not permitted in this study; therefore, direct biomass measurement by harvesting was not conducted. Tree biomass was instead estimated using species-specific allometric equations derived from previous destructive sampling studies, combined with field-measured DBH. We adopted the allometric equation developed by Chen et al. (2025) for young *Catalpa bungei* plantations (Chen et al., 2025). This model was established based on destructive sampling of 3–8-year-old stands across multiple sites in Shandong, Henan, and Hubei provinces, which closely match the age structure and ecological conditions of the present study area. The aboveground biomass was estimated as shown in Equation 1.

(1) $$W=e^{-2.313}\;DBH^{2.368}$$

Where *W* is the biomass of aboveground.

### 2.3 Methods

#### 2.3.1 Correlation analysis

Correlation analysis refers to the examination of two or more interrelated variable elements to assess the strength and direction of their relationships. Before constructing the model, Pearson correlation analysis was conducted using SPSS software on individual tree structural factors obtained via LiDAR: *L_CD_*, *L_H_*, and *AGB*.

#### 2.3.2 Base models

Based on previous studies, five commonly used models were selected as base models for AGB estimation (Fu et al., 2020; Xie et al., 2023): Logistic, Linear, Exponential and Power models (Equations 2-5). These models were fitted to the data to determine the optimal base model for constructing an NLME model.

(2) $$AGB\;=\;\frac{\beta_{1}}{1\;+\;\beta_{2}\;\exp(-\beta_{3}\;L_{H}-\beta_{4}\;L_{CD\;})}\;+\;\epsilon$$

(3) $$AGB\;=\;\beta_{1}\;L_{H\;}\;+\;\beta_{2}\;L_{CD\;}+\;\beta_{3}\;+\;\epsilon$$

(4) $$AGB\;=\;\beta_{1}\;\exp\;(-\beta_{2}\;L_{H}\;-\beta_{3}\;L_{CD})\;+\;\epsilon$$

(5) $$AGB\;=\;\beta_{1}\;(L_{H}^{\beta_{2}})\;(L_{CD}^{\beta_{3}})\;+\;\epsilon$$

Where $\beta_{1}$- $\beta_{4}$*are parameters and ϵ is an error term.*

The best performing model selected based on the following statistical criteria was used for further analyses (Zeng et al., 2021; Zou et al., 2024), as defined in Equations 6–9:

(6) $$R^{2}=1-\sum {\left(AGB_{t}-A\overset{⌢}{G}B_{t}\right)}^{2}/\sum {\left(AGB_{t}-A\bar{G}B_{t}\right)}^{2}$$

(7) $$RMSE=\sqrt{\frac{1}{n}\sum_{i=1}^{n}{\left(AGB_{t}-A\overset{⌢}{G}B_{t}\right)}^{2}}$$

(8) $$ASE=\frac{1}{n}\sum_{i=1}^{n}\left(AGB_{t}-A\overset{⌢}{G}B_{t}\right)$$

(9) $$TRE=\sum (AGB_{t}-A\overset{⌢}{G}B_{t})/\sum A\overset{⌢}{G}B_{t}\times 100$$

Where *AGB_t_* and $A\hat{G}B_{t}$ are the AGB estimated by the allometric equation and predicted by the newly developed AGB model, respectively, and $\bar{AGB}$ is the mean AGB by the allometric equation; *n* is the sample size; and *R²*, *RMSE*, *ASE*, *MSE*, and *TRE* are the coefficient of determination, standard error of estimate, average system error, mean relative error, and total relative error, respectively. Among these, *R*² is the most commonly used in regression models for assessing goodness-of-fit; *RMSE* is defined as the combination of the mean bias and its variance and is the most important evaluation criterion of the model; *ASE* quantifies the systematic bias of the model predictions, representing the mean difference between observed and predicted values; *TRE* and *MSE* are important measures of fitting performance as relative error indices.

#### 2.3.3 Dummy variable model

Dummy variables, also known as indicator variables, are commonly used to handle qualitative factors or categorical variables, typically taking values of 0 or 1 (Zhang et al., 1999). For a qualitative variable *x*, *δ (x, i)* is defined such that *δ (x, i)* = 1 if *x* takes the *i*th category, and 0 otherwise (Gao et al., 2015). In this study, the collected data came from two different density planting cultivation methods. The sample tree in the *i*th region was labeled as *P_i_*. To convert the qualitative data *P_i_* into (0, 1), dummy variables were introduced to represent the different cultivation methods with categorical codes (Equation 10). This approach allowed for the integration of the data into a unified model, thereby reducing workload and enhancing model compatibility (Yang et al., 2015).

(10) $$P_{i}=\{\begin{array}{c} \;1,\text{ high}-\text{density planting}\\ \;0,\text{ low}-\text{density planting} \end{array},i=1$$

Thus, the formulas for incorporating cultivation-type dummy variables into Equations 11-14 are as follows:

(11) $$AGB=\frac{\beta_{1i}P_{i}}{1+\left(\beta_{2i}P_{i}\right)\;exp\;(-(\beta_{3i}P_{i}\;L_{H}\;+\;\beta_{4i}P_{i}\;L_{CD}))}\;+\;\epsilon$$

(12) $$AGB\;=\;\beta_{1i}P_{i}\;L_{H}+\beta_{2i}P_{i}\;L_{CD}+\beta_{3i}P_{i}\;+\;\epsilon$$

(13) $$AGB\;=\;\beta_{1i}P_{i}\;exp(-\beta_{2i}P_{i}\;L_{H}-\beta_{3i}P_{i}\;L_{CD\;})\;+\;\text{ϵ}$$

(14) $$AGB\;=\;(\;\beta_{1i}P_{i})(L_{H}^{\beta_{2i\;}P_{i}})(L_{CD}^{\beta_{3i}\;P_{i}})\;+\;\epsilon$$

Where $\beta_{1i}$, $\beta_{2i}$, $\beta_{3i}$, $\beta_{4i}$ (*i* = 1,2) are the parameter values related to site conditions in the dummy variable model; *P_i_* is a dummy variable; Other parameters are defined as in the base model.

#### 2.3.4 NLME model

The NLME model is closely related to random effects in modeling. Mixed-effects models incorporate both fixed-effect parameters and random-effect parameters in their regression functions (Pinheiro et al., 2015; Fu and Tang, 2020). The general form of a one-level mixed-effects model is shown in Equation 15.

(15) $$\left\{ \begin{array}{c} y_{ij}\;=\;f(\varphi_{i},X_{ij})\;+\;\epsilon_{ij},\;i\;=\;1,\ldots ,\;M,\;j\;=\;1,\ldots ,n_{i},\\ \varphi_{i\;}=\;A_{i}\beta \;+\;\sum_{k=1}^{K}B_{i}^{\left(k\right)}u_{i}^{\left(k\right)},\\ u_{i}^{\left(k\right)}\;∼N\;(\;0,\psi^{\left(k\right)}),cov(u_{i}^{\left(k\right)},u_{i}^{\left(l\right)})=0,\;k\;\neq \;l,\;k\;=\;1,\ldots K,\;l\;=\;1,\ldots K,\\ \epsilon_{i}∼N(0,R_{i}),\;\epsilon \;=\;(\;\epsilon_{i1},\ldots ,\epsilon_{in_{i}})T \end{array}\right.$$

Where *y_ij_* is the univariate response value for the *j*th observation for the *i*th subject; *M* is the number of groups; *n_i_* is the number of observations in the ith group; *f* (*ϕ_i_, X_ij_*) is a nonlinear function describing the relationship between the fixed-effect parameter vector *ϕ_i_* and covariates *X_ij_*; *ϕ_i_* is a specific parameter vector; *A_i_* and *B_i_^(k)^* are design matrices; *β* is a fixed-effect vector of dimension *p0×1*; *u_i_^(k)^* is a *q^(k)^*×1 dimensional random-effect vector for construct k on the *i*th subject; *ψ^(k)^* is the covariance matrix of *u_i_^(k)^*; and *ϵ_ij_* is the error term, assumed to be normally distributed with zero mean and covariance matrix *R_i_*.

In this study, *Catalpa bungei* genotype was used as the random effect. The optimal NLME model was selected by evaluating different combinations of random-effect terms using the Akaike Information Criterion (AIC) and Bayesian Information Criterion (BIC), as shown in Equations 16 and 17. The NLME model was implemented using the “nlme” package in R version 4.4.1.

(16) AIC = 2k - ln(L)

(17) BIC = kln (N) - 2ln(L)

Where *k* is the number of model parameters, *N* is the number of samples, and *L* is the value of the likelihood function.

#### 2.3.5 Determining the structure of the between genotype variance– covariance matrix (Ψ)

The variance–covariance matrix for the random effects at the sub-sample plot level, Ψ, which is common to all genotypes, was assumed to account for the variability of AGB across these plots. Ψ was assumed to be unstructured (Meng and Huang, 2009). We assumed the 2 × 2 variance-covariance matrix as shown below (Equation 18):

(18) $$\Psi \;=\;\left[\;\begin{array}{cc} \sigma_{11}^{2} & \rho_{12}\\ \rho_{21} & \sigma_{22}^{2} \end{array}\;\right]$$

Where *σ^2^_*ij*_* (*i*, *j* = 1, 2, *i* = *j*) is the variance of the *i*th random effect, *ρ_ij_* (*i*, *j* = 1, 2, *i* ≠ *j*) is the covariance between the *i*th and *j*th random effects.

#### 2.3.6 Determining structure of within genotype variance–covariance matrix (R)

During the construction of a mixed-effects model, the number of random-effect parameters significantly affects the convergence of the model. The residual error in the variance-covariance matrix of the NLME model is generally balanced by *R_i_*, as expressed below (Equation 19):

(19) $${\text{R}}_{\text{i}}\;=\;{\text{σ}}_{\text{n}}^{2}\;{\text{G}}_{\text{i}}^{0.5}\;{\text{Γ}}_{i\;}{\text{G}}_{\text{i}}^{0.5}$$

Where $\sigma_{n}^{2}$ is the residual variance (error dispersion factor); *G_i_* is a diagonal matrix representing variance heterogeneity; and $\;\Gamma_{i}$ is the observation matrix of plot *i* with dimension *n_i_*×*n_i_*.

We evaluated two commonly used variance stabilization functions: the exponential function (Equation 20) and the power function (Equation 21) to address variance heterogeneity. Subsequently, the most effective variance function was selected using the likelihood ratio test (LRT) and the Akaike Information Criterion (AIC).

(20) $$\mathrm{var}\left(\epsilon_{ij}\right)\;=\;\sigma^{2}\;\exp\;\left(2\gamma x_{ij}\right)$$

(21) $$\mathrm{var}\;\left(\epsilon_{ij}\right)\;=\;\sigma^{2}\;x_{ij}^{2\gamma }$$

Where *x_ij_* is a selected predictor (*L_H_* and *L_CD_*); and *γ*, *γ_1_*, and *γ_2_* are parameters to be estimated.

To enhance the clarity of model interpretation and avoid potential ambiguities, **Table 2** summarizes the parameter symbols used in the dummy variable model and the NLME AGB model.

**Table 2:** Notation and definitions used in the NLME AGB model.

Symbol	Definition	Type
*P_1_*	Dummy variable for planting density	Categorical predictor
$\beta_{1}$	Scaling coefficient of the power model	Fixed effect
$\beta_{2}$	Exponent of tree height in the base model	Fixed effect
$\beta_{3}$	Density-related adjustment to the height exponent	Fixed effect
$\beta_{4}$	Exponent of canopy diameter in the base model	Fixed effect
*u_1_*	Genotype-specific random effect associated with $\beta_{2}$	Random effect
*u_2_*	Genotype-specific random effect associated with $\beta_{4}$	Random effect
*Ψ*	Variance–covariance matrix of random effects	Random structure
*ϵ*	Residual error term	Error
$R_{i}$	Within-genotype variance–covariance matrix	Error structure

#### 2.3.7 Model estimation

The maximum likelihood with the Lindstrom and Bates (LB) algorithm implemented in the R software (version 4.4.1) nlme function was used to estimate all NLME model variants. Many studies have described the LB algorithm and nlme functions (Bates, 1990; Carey and Wang, 2000).

#### 2.3.8 Subject-specific prediction

The NLME AGB estimation model was used to predict AGB with and without the random effects involved. When random effects are excluded, the model yields the population-average (mean) response, referred to as the M-response. In contrast, incorporating predicted random effects results in a subject-specific (localized) model. This process of integrating random effects into the model is termed calibration.

Calibration requires prior information on the response variable—in this case, AGB measurements obtained from a sub-sample of trees. The random effects were predicted using the empirical best linear unbiased prediction (EBLUP) approach. In scenarios where such prior information is unavailable, AGB prediction may be performed using either the M-response model or an ordinary least squares (OLS) model, the latter being fitted without random effects.

(22) u^i=Ψ^ZiT(R^i+ZiΨ^^ZiT)−1ei=Ψ^ZiT(R^i+ZiΨ^^ZiT)−1[yi−f(β^,ui∗,xi)+Ziui∗]

Where û*_i_* is a q-dimensional vector of the predicted random effects for the *i^th^* genotype (*i* = 1,…, *M*); ${\text{u}}_{i}^{*}$ is a vector of EBLUP for random effects u*_i_*; f (·) is an NLME AGB estimation model; $\hat{\beta }$ is a vector of the estimated fixed-effect parameters β; *x_i_* is a vector of the predictors; $\hat{\Psi }$ is an estimated variance-covariance matrix for the random effects u*_i_* (*i* = 1,…, *M*); $\hat{{\text{R}}_{î}}$ is an estimated variance-covariance matrix for the error term e*_i_*; *Z_i_* is an *n_i_* × *q* dimensional design matrix of partial derivatives of NLME AGB estimation model f (·) with respect to random effects u*_i_*. As the unknown random effects appeared on both sides of Equation 22, no direct algebraic solution for û*_i_* is possible. However, for its algebraic solution, Meng and Huang developed a three-step iterative algorithm based on the EBLUP theory for the prediction of random effects (Meng and Huang, 2009). The computer program using R software for this algorithm was presented by Fu et al (Fu et al., 2017).

We employed AGB measurements from varying numbers of sample trees to predict the random effects in the model. In general, prediction accuracy improves with an increasing number of samples used for model localization. Numerous modeling studies have sought to determine the optimal sample size required to calibrate NLME models while maintaining reliable predictive performance. Based on previous research, we implemented the following four sampling strategies for selecting trees in each genotype to account for subject-specific variability in the response variable (AGB):

- AGB of 1–10 smallest trees per genotype.
- AGB of 1–10 largest trees per genotype.
- AGB of 1–10 medium-sized trees per genotype, randomly selected from those with sizes between the 30th and 70th percentiles.
- AGB of 1–10 randomly selected trees per genotype, regardless of size.

The prediction performance of each strategy using 1 to 10 sample trees was evaluated using commonly adopted metrics: total relative error (TRE) and standard error of estimation (ASE). Each sampling strategy was repeated 100 times to ensure robustness. The AGB of the remaining trees in each genotype was estimated by averaging the predictions derived from the repeated sampling results.

#### 2.3.9 Model evaluation

We employed leave-one-genotype-out cross-validation (LOOCV) to evaluate the performance of the nlme model. In each iteration, one sample of a particular genotype was assigned as the validation set, while the remaining samples from all other genotypes constituted the training set. The AGB model was fitted using the training data to estimate model parameters. These parameter estimates were then used to generate predictions for the validation set, and corresponding prediction statistics were computed. After validating against one genotype, the corresponding sample was returned to the data set, and the procedure was repeated with another genotype serving as the new validation set. This process was iterated until every distinct genotype had been used exactly once as the validation set.

## 3 Results

### 3.1 Correlation analysis

In this study, the Pearson correlation coefficients between *AGB*, *L_CD_* and *L_H_* were 0.77, and 0.79, respectively—all of which passed the 0.01 significance level. This indicates that *AGB* has a strong correlation with *L_CD_* and *L_H_*. Therefore, these two variables can be used as independent variables in model construction.

### 3.2 Selection of the base model

Both Equations 2 and 5 showed a slightly superior fitting performance compared to Equations 3 and 4 (**Table 2**). Relative to Equation 2, Equation 5 is more simplified with only four parameters, and it was therefore chosen as a basic nonlinear model to build the generalized NLME AGB estimation models based on the LiDAR-derived predictors. The estimated parameters for Equations 2-5 are listed in **Table 3**.

**Table 3:** Statistical fitting results of base AGB models.

Model	Number of parameters	Evaluating indicator					
		RMSE (kg)	R²	TRE	ASE	AIC	BIC
Logistic	4	4.3345	0.7154	16.6789	-0.1289	16982	17012
Linear	3	4.7202	0.6625	19.7791	0.0000	17481	17505
Exponential	3	4.5791	0.6824	18.6146	-0.4749	17303	17327
Power	3	4.3377	0.7150	16.7039	-0.1296	16984	17008

### 3.3 Dummy variable model construction

We simplified the form of the dummy variable model to improve the success rate of fitting the NLME model. The three extended equations are as follows:

(23) $$AGB\;=\;(\beta_{1\;}+\;\beta_{2}\;P_{1})\;L_{H}^{\beta_{3}}\;L_{CD}^{\beta_{4}}\;+\;\epsilon$$

(24) $$AGB\;=\;\beta_{1}\;L_{H}^{\left(\beta_{2}+\beta_{3}\;P_{1}\right)}\;L_{CD}^{\beta_{4}}\;+\;\epsilon$$

(25) $$AGB\;=\;\beta_{1}\;L_{H}^{\beta_{2}}\;L_{CD}^{\left(\beta_{3}+\beta_{4}\;P_{1}\right)}\;+\;\epsilon$$

Compared with the basic power model (Equation 5), the power model incorporating dummy variables (Equation 24) showed consistently better predictive performance. Allowing model parameters to vary across density groups improved the model’s ability to represent structural heterogeneity in tree AGB. Specifically, the dummy-variable model reduced RMSE by 9.68%, increased R² by 7.34%, and decreased TRE by 18.42% relative to the conventional power formulation. In addition, both AIC and BIC were substantially lower for the dummy-variable model, indicating that the improvement in goodness-of-fit was achieved without an excessive increase in model complexity. Overall, introducing density-related dummy variables provided a more reliable and interpretable framework for AGB estimation. The estimated parameters for Equations 23-25 are summarized in **Table 4**.

**Table 4:** Fitting statistics of the dummy variable AGB models.

Model	Number of parameters	Evaluating indicator					
		RMSE (kg)	R²	TRE	ASE	AIC	BIC
Power+Dummy (24)	4	3.9246	0.7667	13.6737	-0.0497	16395	16419
Power+Dummy (25)	4	3.9179	0.7675	13.6268	-0.0604	16385	16409
Power+Dummy (26)	4	3.9263	0.7665	13.6854	-0.0626	16398	16422

### 3.4 Construction of the NLME model

**Figure 3:**
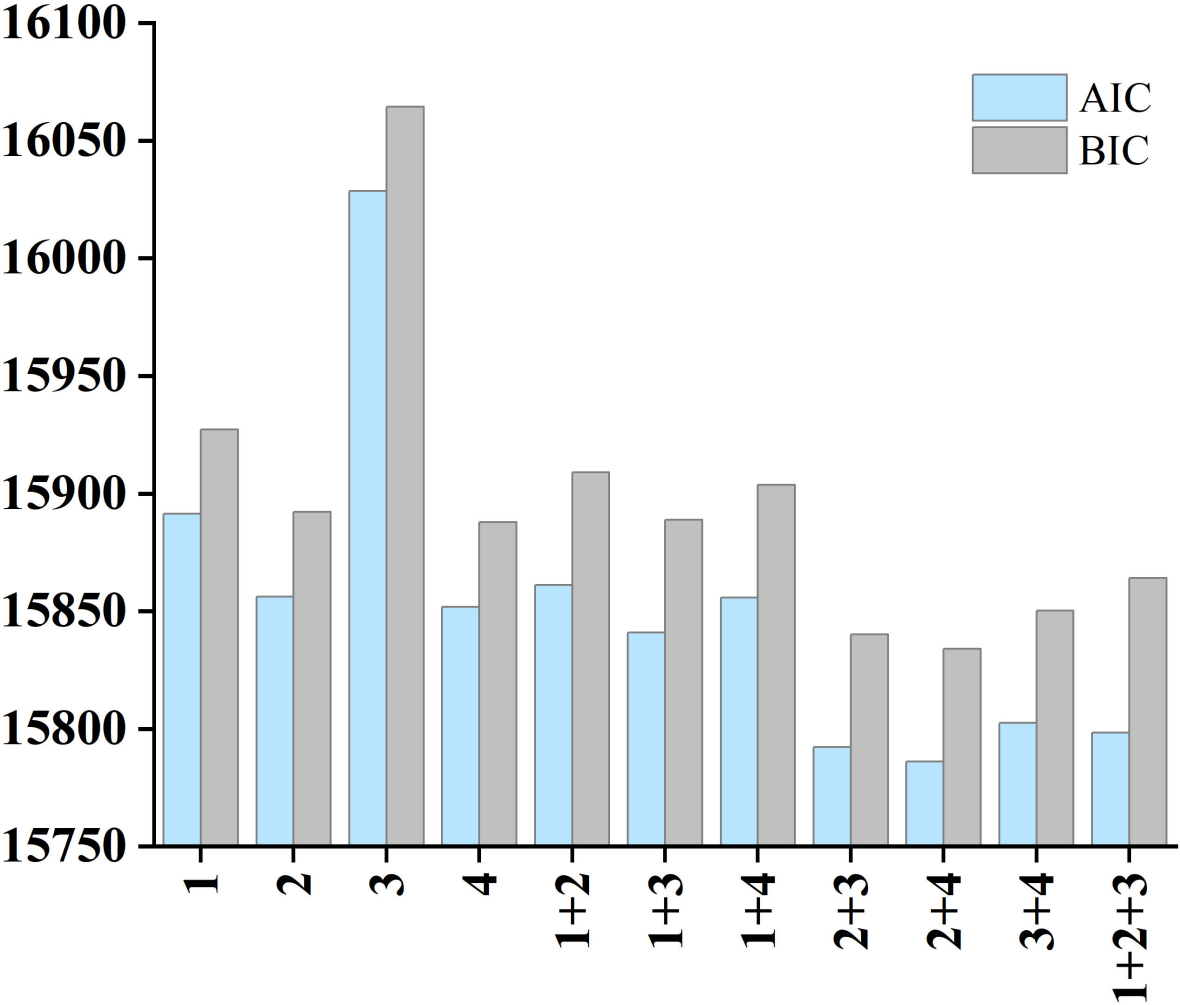
The 11 combinations of random effects for dummy variable model (25) (numbers 1~4 represent parameters included as random effects; “+” indicates combination of random effects; with blue and gray bars representing AIC and BIC values, respectively, and lower values indicating better model performance).

Considering the four parameters in the model, there were 11 feasible combinations of random effects in the base model. All NLME models successfully converged. The best model, based on the minimum AIC (15786) and BIC (15833), corresponded to the combination of parameters 2 and 3, as shown in **Figure 3**.

The NLME model exhibited consistently improved predictive performance compared with the power function model with dummy variables. Specifically, the NLME framework reduced the RMSE by 2.07% (from 3.9179 kg to 3.8370 kg), increased R² by 8.31% (from 0.7675 to 0.8313), and decreased the TRE by 3.97% (from 13.6268% to 13.0858%). In addition, the NLME model yielded substantially lower AIC and BIC values than the dummy-variable model, indicating a markedly improved balance between model goodness-of-fit and complexity. These results demonstrate that incorporating random effects within the NLME framework more effectively captures genotype level heterogeneity and enhances the robustness and accuracy of individual-tree AGB prediction.

(26) $$AGB\;=\;\beta_{1\;}\;L_{H}^{\left(\beta_{2}\;+\;u_{1}\right)\;+\;\beta_{3}\;P_{1}}\;L_{CD}^{\left(\beta_{4}\;+\;u_{2}\right)}\;+\;\epsilon$$

Where $\beta_{1}$- $\beta_{4}$ are fixed effect parameters; *u_1_*and *u_2_* are the random effects of *Catalpa bungei* genotypes on parameters $\beta_{2}$ and $\beta_{3}$; *P_1_* is a dummy variable; and *ϵ* is the error term.

**Figure 4:**
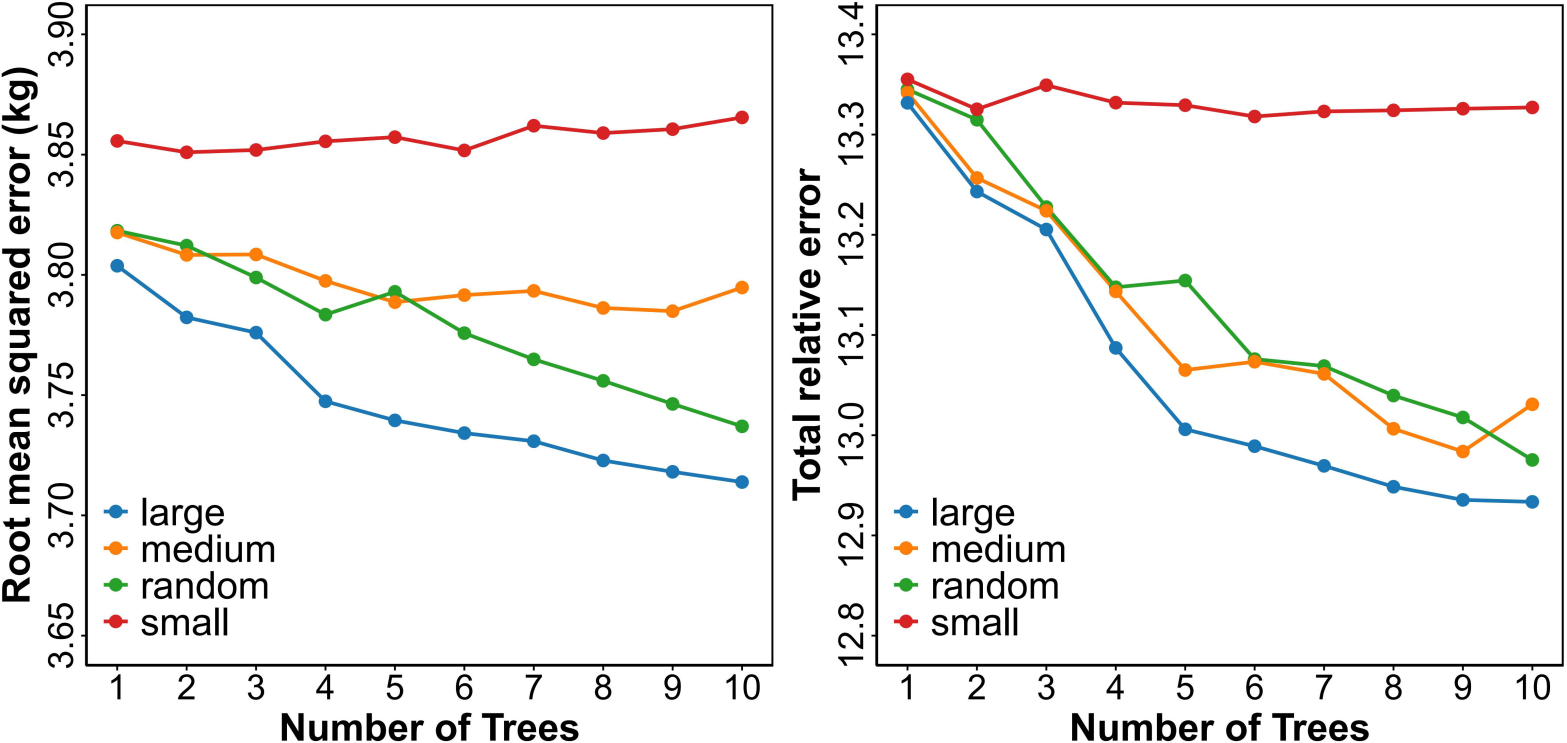
Calibration of AGB using Equation 27 under four sampling strategies and different sample sizes, showing RMSE and TRE for the estimated random effects across genotypes. Sampling strategies include: Random–randomly selected trees; Maximum–largest trees; Medium–medium-sized trees; and Minimum–smallest trees.

The estimated parameters for the NLME AGB model with dummy variables (Equation 26) are summarized in **Table 3**. All fixed-effect coefficients reached statistical significance (p< 0.05). Diagnostic analysis of the residuals, however, revealed persistent heteroscedasticity in Equation 26 even after the inclusion of random effects (**Figure 4a**). Meanwhile, evaluation of the empirical autocorrelation function indicated no significant temporal dependencies among the standardized residuals across genotypes.

### 3.5 Parameter estimation

We evaluated two variance-stabilizing functions, and the power variance function with *L_CD_* as a predictor showed the best performance (**Table 5**), and therefore it was applied in Equation 26. All the estimates of Equation 26, with the power variance function included along with the evaluation metrics, are listed in **Table 6**. After substituting the estimated value of the fixed effects parameter into Equation 26, the final NLME AGB estimation model for *Catalpa bungei* a would become:

**Table 5:** Assessment of the NLME AGB estimation model (26) with different variance functions (EF, exponential function – Equation 20; PF, power function – Equation 21; and variance function 1 meant that the variances were homogeneous).

Variance	*L_H*				*L_CD*			
Function	AIC	-2LL	LR	p_value	AIC	-2LL	LR	p_value
1	15786	15770			15786	15770		
EF	13476	13458	2318	<0.0001	13211	13193	2584	<0.0001
PF	12922	12904	2873	<0.0001	12545	12527	3249	<0.0001

**Table 6:** Parameter estimates and fit statistics of four different models (AIC, Akaike’s information criterion; and LL, log-likelihood).

	Parameters	Model (5)	Model (24)	Model (26)	Model (27)
Fixed-effects parameters	$\beta_{1}$	0.3918	0.1519	0.1417	0.0733
	$\beta_{2}$	1.3996	2.2562	2.3008	2.8351
	$\beta_{3}$	1.0420	-0.2878	-0.3039	-0.3437
	$\beta_{4}$		0.7550	0.7461	0.4963
Variance components	$s_{1}$			0.0672	0.0653
	$s_{2}$			0.1713	0.1807
	$s_{12}$			-0.1029	-0.1032
	γ				1.4753
	σ	4.3392	3.7475	3.4007	1.0684
Model performance	AIC	16984	16384	15786	12545
	-2LL	16976	16377	15770	12527

(27) $$\begin{array}{l} AGB\;=\;0.0733\;\cdot \;L_{H}^{\left(2.8351\;+\;u_{1}\right)\;+\;\left(-0.3437\;\cdot \;P_{1}\right)}\cdot L_{CD}^{\left(0.4963\;+\;u_{2}\right)}\;+\;\epsilon \\ u_{i}\;=\;\left[\begin{array}{c} u_{1}\\ u_{2} \end{array}\right]∼\;N\;\{\left[\begin{array}{c} 0\\ 0 \end{array}\right],\psi_{1}\;=\;\left(\begin{array}{cc} 0.06527 & -0.1032\\ -0.1032 & 0.1807 \end{array}\right)\}\\ \epsilon_{i}\;=\;{\left(\;\epsilon_{i1},\ldots ,\epsilon_{in_{i}}\;\right)}^{T}∼\;N\;\left(0,\;R_{i}\;=\;1.14146\;\cdot \;G_{i}^{0.5}\;\Gamma_{i}\;G_{i}^{0.5}\;\right)\\ G_{i}\;=\;diag\;\left(L_{CDi1}^{2.951},\ldots ,L_{CDin_{i}}^{2.951}\right)\\ \Gamma_{i}\;=\;I_{n_{i}} \end{array}$$

Equation 27 led to a smaller AIC and larger LL values than both Equations 5, 24 and 26 with homogeneous error variances, indicating that the sample sub-sample genotypes exerted significant random effects on the predictions of AGB.

### 3.6 Subject-specific AGB prediction

The predictive statistics for calibrated responses using different sampling strategies are illustrated in **Figure 4**. Both the RMSE and TRE for the four sampling strategies exhibited similar trends. Regardless of the number of trees from one to ten trees used for calibration at the sample genotype level, both RMSE and TRE showed consistent patterns across strategies. Calibration with the largest trees demonstrated a clear accuracy advantage. When the sample size increased to four trees, TRE showed the greatest reduction to 13.09%, representing a further reduction from the two-tree sample that achieved a TRE of 13.24%. The model’s ability to characterize canopy structural heterogeneity also improved, with RMSE decreasing from 3.80 kg to 3.75 kg. For large-scale forest inventory needs, the two-tree largest-tree calibration plan with a TRE of 13.24% provides a cost-effective solution, reducing error compared to a single-tree sample that had a TRE of 13.33%, while meeting the accuracy requirements of routine monitoring. The inclusion of additional sample trees could improve accuracy but would increase inventory costs. The model quantified the nonlinear modulating effects of environmental factors through the interaction parameters between *L_CD_* and *L_H_*, and significantly alleviated heteroscedasticity by applying a power variance function. This resulted in improved uniformity in residual distribution. This study proposes a tiered sampling framework. For high-precision requirements, the use of four trees with the largest AGB achieving a TRE of 13.09% is recommended, while two trees with a TRE of 13.24% are sufficient for routine monitoring. This provides a scientific and operational approach for AGB estimation in multi-genotype *Catalpa bungei* stands.

### 3.7 Model evaluation

The prediction statistics based on the cross‐validation method for Equation 5, Equations 24, 27 in two cases, at the M response and genotype level, are presented in **Table 7**.

**Table 7:** Prediction statistics of three models using the leave-one-genotype-out cross-validation (M response, mean response).

Model	RMSE (kg)	R²	TRE	ASE
Model (5)	4.3550	0.7127	16.8374	-0.1284
Model (24)	3.9664	0.7651	13.5935	-0.0548
Model (27)				
M response	3.8530	0.7751	13.1793	0.0432
Genotype level	3.7095	0.7916	12.9161	0.0319

During cross-validation, the calibrated NLME model d Equations 27 demonstrated the best predictive performance for individual tree AGB, achieving the highest R² of 0.7916 and the lowest RMSE of 3.7095. This indicates that the inclusion and calibration of random effects significantly improved the model’s prediction accuracy.

Compared to the basic power function model Equations 5, the calibrated NLME model increased R² by 11.07% and reduced RMSE by 14.82%. It also outperformed both the non-calibrated NLME model (M response) and the power model with a dummy variable Equation 24. The results confirm that incorporating and calibrating random effects at the sub-sample level substantially enhanced the prediction accuracy. Therefore, the calibrated form of Equation 27 is recommended for predicting individual tree AGB using LiDAR-derived metrics.

**Figure 5:**
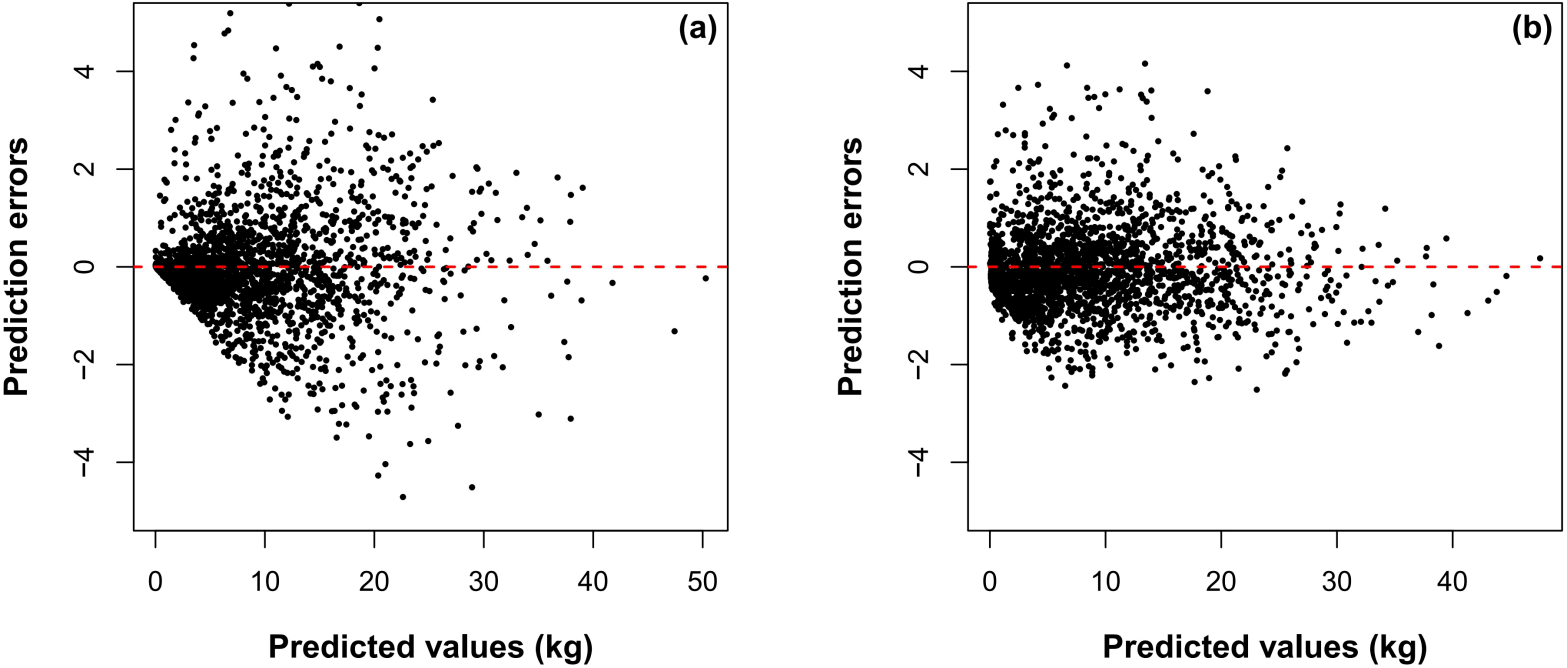
Prediction errors based on Equation 26 with a variance-stabilizing function excluded **(a)** and prediction errors based on Equation 27**(b)** with a variance-stabilizing function included.

**Figure 5b** shows the residuals distribution of the NLME AGB estimation model (Equation 27) at the genotype level based on the leave-one-genotype-out cross-validation. Compared to Equation 26 with homogeneous error variances (**Figure 5a**), heteroscedasticity was significantly reduced for Equation 27. This showed that the power variance function applied with *L_CD_* as a predictor effectively accounted for heteroscedasticity (**Figure 5b**).

### 3.8 Model application

**Figure 6:**
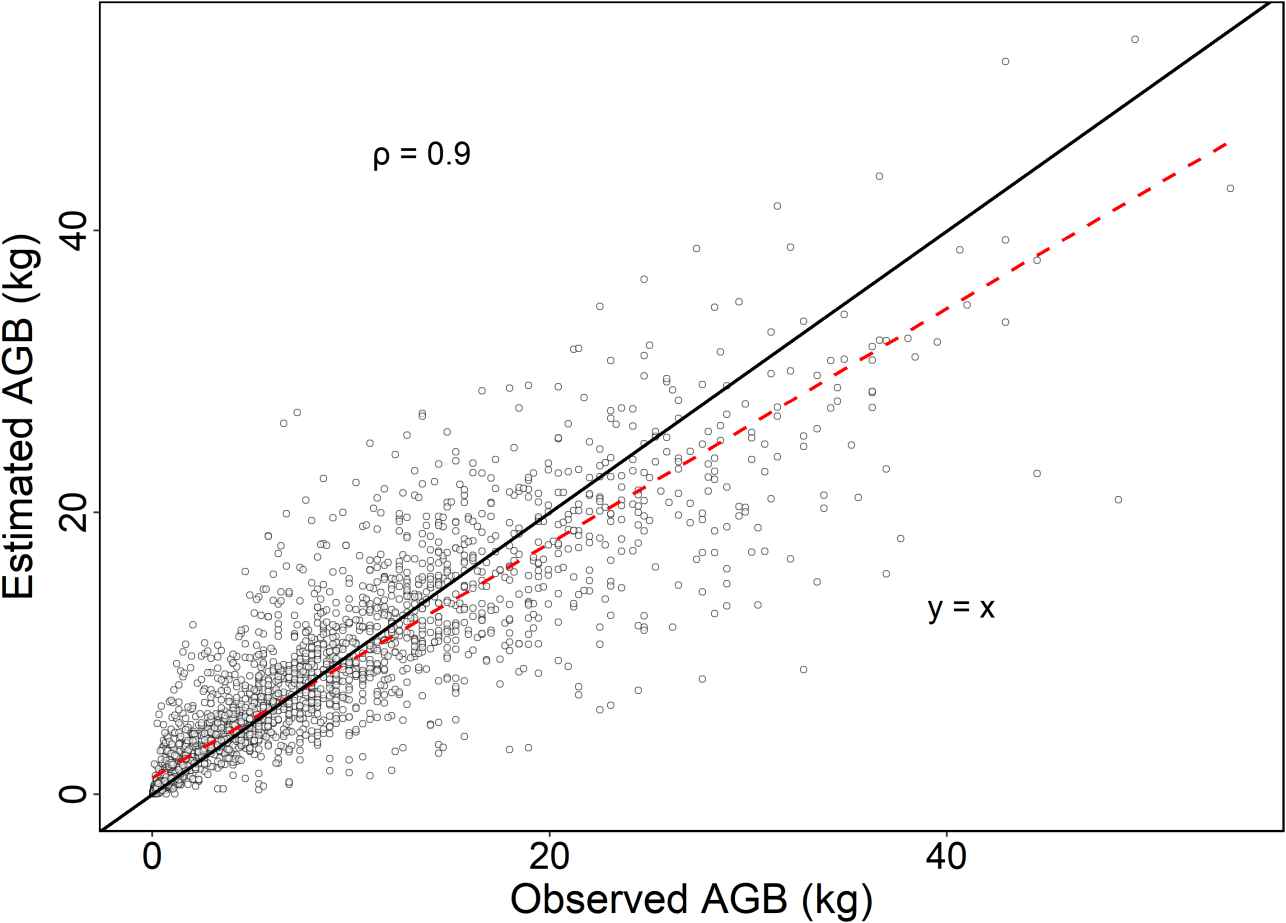
Scatter plot of the observed AGB against that from the estimated by Equation 27, with the red dotted line illustrating a linear relationship between two variables, the black line denoting y = x, *ρ* is the correlation coefficient of the estimated AGB by Equation and the observed AGB.

**Figure 6** shows the scatter plot of the predicted AGB against that by Equation 27. This figure confirmed that the observed AGB and NLME AGB estimates were very close and the corresponding Pearson correlation coefficient of the two sets of AGB estimates was 0.9. This suggested that the presented NLME AGB estimation model could be used for precise estimation of AGB, and is thus important for informed decision-making in forestry.

## 4 Discussion

### 4.1 Core findings and advantages of the NLME approach

In this study, an NLME model was developed using *L_H_*, *L_CD_*, and field-measured data to establish an AGB prediction model for *Catalpa bungei*. The results show that the calibrated NLME model provided improved predictive performance compared with the basic power function model, achieving an R² of 0.7916 and an RMSE of 3.7095 kg, suggesting an improved ability to characterize the nonlinear relationships between tree height, crown width, and AGB. Compared with the uncalibrated power function model (Equation 5), the calibrated NLME model reduced RMSE by 14.82% (from 4.3550 kg to 3.7095 kg) and decreased the TRE by 23.29% (from 16.84% to 12.92%). These improvements indicate that incorporating and calibrating random effects at the genotype level substantially enhanced prediction accuracy. In addition, the relatively uniform distribution of residuals further supports the reliability of the NLME framework. To account for heteroscedasticity in the AGB data, a variance power function structure (var Power) was implemented, with *L_CD_* specified as the variance covariate. The estimated power parameter was 1.475, indicating that residual variance increased proportionally with *L_CD_*^1.475. This variance structure effectively stabilized residual dispersion across tree size classes and improved the robustness of parameter estimation, particularly for larger trees.

In recent years, UAV-based LiDAR has been increasingly used for single-tree scale forest parameter estimation due to its high vertical resolution and its capability to characterize three-dimensional canopy structure using dense point clouds (Hu T. et al., 2021). As an active remote sensing technology, LiDAR records the timing and intensity of laser returns, enabling consistent measurement of tree height and crown-related attributes under varying illumination conditions, particularly when compared with passive optical imagery. In the study area, *Catalpa bungei* trees were artificially planted in 2020, and the sample plots exhibited minimal variation in topographic factors such as elevation, slope, and aspect. Differences among plots were mainly associated with planting density, fertilization treatment, and genotype. The crown architecture of *Catalpa bungei* was well delineated in the LiDAR point cloud, allowing reliable identification of both trunk and canopy outlines and the derivation of height- and crown-related metrics. Under these relatively uniform site conditions, variation in AGB was primarily reflected by differences in vertical growth and crown development. Accordingly, LiDAR-derived tree height and crown width were selected as key predictors for AGB estimation in this study. Similar results have been reported in previous studies, in which tree height and canopy-related LiDAR metrics were identified as important predictors of individual-tree AGB (Li et al., 2025).

### 4.2 Rationale for including a planting-density dummy variable

Planting density is an important management factor affecting individual-tree growth and biomass allocation in plantations. Incorporating planting density as a dummy variable significantly improved model performance, indicating that density-induced structural differences cannot be fully captured by tree height and crown width alone (Chen et al., 2017). High-density stands tend to favor height-dominated growth, whereas low-density stands allow greater crown development, resulting in distinct biomass–structure relationships. By allowing key model parameters to vary between density classes, the dummy-variable formulation effectively accounted for this management-driven heterogeneity and reduced systematic estimation bias. Overall, introducing planting density as a dummy variable provides a biologically meaningful and statistically efficient improvement to individual-tree AGB modeling (Widagdo et al., 2020).

### 4.3 Interpretation of genotype-related differences in AGB

Differences in AGB observed among *Catalpa bungei* genotypes in this study are most plausibly explained by true genetic variation in growth performance and tree architecture. Genetic effects commonly manifest through systematic differences in diameter growth, height development, and crown structure, which are primary drivers of biomass accumulation. In *Catalpa bungei*, clone/genotype trials have documented significant genotypic variation and genotype-by-environment effects in growth traits, supporting the interpretation that persistent AGB differences among genotypes can reflect genuine genetic signals (Xiao et al., 2019). Moreover, in plantation trials, competitive ability and spacing/density can modulate how genetic differences are expressed, meaning that genotype-related differences may become more evident under certain competition regimes. Because all genotypes were evaluated under the same AGB estimation framework and within comparable site and management conditions, the observed AGB differences are unlikely to be driven primarily by environmental heterogeneity and are more plausibly interpreted as genetic effects expressed through measurable, LiDAR-observable structural traits.

From a statistical perspective, genotype-level random effects in the NLME framework should not be interpreted as direct measurements of genetic effects, but rather as statistical proxies for genetically driven variation expressed through observable phenotypic traits. Under uniform site and management conditions, these random effects capture persistent deviations in biomass–structure relationships that reflect the integrated expression of genetic potential and phenotypic plasticity (i.e., genotype × phenotype integration) (Li et al., 2017). Compared with plot- or site-level random effects, genotype-level random effects are more consistent with the biological hierarchy of multi-genotype experimental plantations and reduce the risk of confounding genetic signals with local environmental noise. Therefore, in this study, genotype random effects provide a biologically meaningful and statistically robust representation of genetic heterogeneity rather than unmeasured environmental variation.

### 4.4 Methodological implications and calibration strategy

This study established a NLME model with genotype as a random effect and introduced planting density as a dummy variable. Through comparisons of different modeling methods, the optimal AGB inversion model was identified. In constructing the NLME model, the power function consistently showed the best and most stable fit. Compared with fixed-effects models, the mixed-effects model achieved the highest fitting accuracy and more precise AGB estimates. Overall, nonlinear models demonstrated better fit and stability than linear regressions, aligning with previous findings (Chen et al., 2022). AGB modeling approaches vary significantly across studies in terms of the number of predictor variables, sample size, and modeling methods. This study overcomes this limitation by introducing a mixed-effects model and treating the genotype as a random effect. NLME models are particularly suited for forest stands or sample plots with hierarchical structures, capturing stand-level characteristics through fixed effects while quantifying variation at multiple hierarchical levels via random effects (Zhang et al., 2025). Their advantages lie in effectively integrating multisource data, reducing heterogeneity effects, and supporting local calibration using limited samples (Fu et al., 2012, 2013), thus providing a core modeling method for dynamic forest resource monitoring using technologies such as UAV-LiDAR. In constructing NLME AGB models, plot-level and individual tree characteristics are commonly selected as random effect factors to account for heterogeneity and improve predictive performance. Plot-level random effects can reflect AGB variation due to factors such as soil properties, terrain, and light conditions, while tree-level random effects account for heterogeneity in individual tree growth conditions, such as tree age and health status (Xu et al., 2015; Lu et al., 2022; Xie et al., 2023). The inclusion of random effects not only enhances model flexibility but also improves the model’s ability to capture complex patterns in the data, thereby increasing the accuracy of AGB predictions for tree species.

This study also proposed a tiered sampling framework, in which four trees with the largest AGB are used for calibration in high-precision scenarios, while a simplified calibration scheme using two trees with the largest AGB is suitable for routine monitoring. This pattern likely reflects the dominant contribution of larger trees to overall AGB variability, as their AGB provides stronger leverage for capturing genotype-specific AGB–structure relationships during calibration. This strategy achieves a good balance between cost control and accuracy requirements, providing a scientific and flexible solution for AGB estimation in multi-genotype *Catalpa bungei* stands. It highlights the crucial importance of selecting appropriate sample sizes and sampling strategies to enhance model predictive performance and practical applicability. The research shows that sampling schemes and sampling accuracy are influenced by numerous factors such as stand type, plot type, and species type, which in turn lead to differences in research results. Bronisz et al. found that extracting the tree with the largest diameter in each plot resulted in the highest accuracy (Bronisz and Mehtätalo, 2020). Ciceu et al.’s research indicated that extracting an average of six trees had the highest prediction accuracy (Ciceu et al., 2020). In practical applications, there are differences in research locations and tree species, and the sampling methods and sample quantities are difficult to be unified. Therefore, it is necessary to compare different sampling methods and sample quantities in the model to achieve the best prediction effect.

### 4.5 Limitations and model transferability

The UAV LiDAR–based AGB model developed in this study was calibrated using data from a single experimental plantation characterized by controlled site conditions and a specific management regime. While this design ensured internal consistency and allowed detailed analysis of genotype-related AGB variation, model performance may vary when applied to plantations with different environmental settings, stand ages, or silvicultural practices. AGB–structure relationships are influenced not only by canopy architecture captured by LiDAR, but also by site-specific factors such as climate, soil conditions, and stand development history, which may alter growth trajectories and AGB allocation patterns across regions or age classes. As a result, models developed under uniform experimental conditions should not be directly transferred to contrasting environments without recalibration (Wu et al., 2025).

A key feature of the present study is the explicit incorporation of genotype as a random effect within a nonlinear mixed-effects framework. This structure provides a practical mechanism for recalibrating AGB–structure relationships when applied to new sites, and such recalibration can be achieved using a limited number of representative trees per genotype, thereby reducing the need for extensive destructive sampling (Korhonen et al., 2019). Nevertheless, the absence of external validation using independent datasets from different regions or developmental stages represents a limitation of the current study. Future work should evaluate the proposed framework across multiple sites and age classes to better assess its general applicability and to refine genotype-aware AGB models for broader operational use. Despite this limitation, the modeling strategy presented here offers a flexible and efficient basis for extending UAV LiDAR–based individual-tree AGB estimation beyond a single experimental plantation.

## 5 Conclusion

This study successfully achieved high-precision AGB estimation for *Catalpa bungei* by constructing an NLME model that integrates airborne LiDAR data and ground measurements. The model used *L_H_* and *L_CD_* as core predictors, included genotype as a random effect, and optimized the covariance structure, significantly improving prediction accuracy (R² = 0.7916, RMSE = 3.7095). Compared with traditional dummy variable models, the NLME model reduced the TRE by 13.59% to 12.92%, showed residuals evenly distributed around zero, and successfully captured genotype-related heterogeneity. In addition, when calibrated using four trees with the largest AGB, TRE was reduced to 13.09%. A simplified scheme using two largest trees (TRE = 13.24%) provided a cost-effective balance between precision and resource investment, offering a feasible tiered sampling framework for AGB estimation in multi-genotype *Catalpa bungei* stands. The findings indicate that the use of UAV-LiDAR–derived structural metrics within an NLME modeling framework provides a highly accurate approach for remote sensing–based AGB estimation of multi-genotype *Catalpa bungei*. Notably, this study demonstrates that treating genotype as a random effect can explain relatively significant AGB variability attributable to genetic differences—an aspect rarely considered in previous AGB estimation models. This underscores the novel contribution of our framework, as it explicitly incorporates genetic heterogeneity into remote sensing-based AGB modeling.

In addition, the proposed tiered sampling strategy provides a cost-effective and scalable solution. The study demonstrates that measuring only the two largest trees per genotype is sufficient for calibration, substantially reducing fieldwork compared with conventional sampling methods while maintaining high accuracy. This contribution is particularly valuable for operational forest monitoring, where minimizing labor and costs is essential. Overall, this study not only advances methodological innovation by integrating UAV LiDAR with genotype-based mixed-effects modeling but also provides practical solutions for reducing sampling costs. These innovations enhance the applicability of remote sensing in precision forestry, carbon sink assessment, and ecological monitoring.

## Data Availability

The datasets presented in this article are not readily available because the data is restricted due to confidentiality issues; therefore, the dataset cannot be shared. Requests to access the datasets should be directed to QC, chengqiqo@163.com.
